# Metabolic and immune response to high-fat diet in healthy urban Indonesian males with family history of type 2 diabetes mellitus

**DOI:** 10.1900/RDS.2023.19.51

**Published:** 2023-06-30

**Authors:** Akterono Budiyati, Dyah Purnamasari, Heri Wibowo, Indah Suci Widyahening, Pradana Soewondo

**Affiliations:** 1 Doctoral Program in Biomedical Science, Faculty of Medicine, Universitas Indonesia, Jakarta, Indonesia,; 2 Stem Cell and Cancer Institute, Jakarta, Indonesia,; 3 Division of Endocrinology, Metabolism, and Diabetes, Departement of Internal Medicine, Dr. Cipto Mangunkusumo National Referral Hospital, Faculty of Medicine Universitas Indonesia, Jakarta, Indonesia,; 4 Integrated Laboratory, Faculty of Medicine, Universitas Indonesia, Jakarta, Indonesia,; 5 Department of Parasitology, Faculty of Medicine, Universitas Indonesia, Jakarta, Indonesia,; 6 Department of Community Medicine, Faculty of Medicine Universitas Indonesia,; 7 Southeast Asian Ministers of Education Regional Center for Food and Nutrition (SEAMEO RECFON) Pusat Kajian Gizi Regional Universitas Indonesia.

**Keywords:** type 2 diabetes mellitus, high-fat diet, insulin resistance, TNF-a/IL-10 ratio, first-degree relatives

## Abstract

**Objectives:**

Non-diabetic first-degree relatives (FDR) of type 2 diabetes mellitus (T2DM) patients have been reported to have relatively higher insulin resistance and inflammatory markers compared to population without family history of T2DM. We investigated whether healthy FDR T2DM of Indonesian males living in urban area are more susceptible to the adverse effects of high-fat diet (HFD) than non-FDR subjects.

**Methods:**

Twentyseven normoglycemic and normotensive FDR males and 28 ageand- body-mass-index-(BMI)-matched healthy non-FDR males underwent a 5-days HFD challenge. Dietary intake before and after HFD were collected by 24-hours food recall. Metabolic profiles and plasma cytokine levels were assessed before and after the HFD intervention.

**Results:**

Within similar BMI profile between groups, FDR subjects showed significantly bigger waist circumference (p=0.001) and higher triglyceride (p=0,03) than those of non-FDR. Despite similar HOMA-IR and IL-6 responses to 5-days HFD, significant increase of plasma TNF-α/IL-10 ratio found in FDR subjects, while in contrary, TNF-α/IL-10 ratio significantly decreased in non-FDR group (p<0.001), resulting an OR of 7.1 (95% CI 2.2-23.4) for FDR to develop elevated plasma TNF-α/IL-10 ratio in response to HFD. The tendency was as high as 24.8 (95% CI 2.3-262.6) in FDR subjects with BMI ≥25 compared to the corresponding non-FDR subjects.

**Conclusions:**

High-fat diet induced insulin resistance and increase of IL-6 plasma in healthy adult Indonesian males. Immune response polarization favouring proinflammatory environment was predominantly occurred in FDR subjects when compared to those of non- FDR subjects. Alteration of lipid accumulation was highly likely contributed to greater HFD-inflammation effects on FDR than non-FDR subjects.

## 1 Introduction

Individuals with a family history of type 2 diabetes mellitus (T2DM) are known to have a higher risk of developing insulin resistance (IR) and later on T2DM, especially when living in the obesogenic environment, such as excessive caloric intake and sedentary lifestyle [1]. This risk is suggested to be associated with their smaller energy storage capacity in adipose tissue, so that as a consequence of obesogenic environment, they are more prone for having lipid accumulation in ectopic tissue, such as visceral adipose tissue (VAT), skeletal muscle tissue, and liver compared to the non-FDR subjects [1,2].

Excessive lipid accumulation in cells can interfere with insulin signalling causing improper response to insulin action and lead to IR [2]. Several population-based studies support this hypothesis by providing evidences that subclinical characteristic pertaining to IR, such as central obesity and dyslipidemia, were frequently occurred within healthy first-degree relatives (FDR) of T2DM subjects [1,3,4] particularly within young age (16-24 years old) [5].

Excessive ectopic lipid accumulation is also accompanied by increased production of some proinflammatory molecules, such as TNF-α, IL-1β, and IL-6 and known as systemic low-grade chronic inflammation which increased individual risk to atherosclerosis [1,6]. In addition to metabolic alteration, increased level of circulating IL-1β [7] and TNF-α [8] were also found within FDR subjects compared to the non-FDR, suggesting the multiple risks factors might correlate FDR susceptibility to IR and T2DM.

Experimental models using high-fat diet (HFD) to create conditions that mimic excessive caloric intake are often used to reveal the metabolic deviations related to IR that has already occurred at early stage in FDR subjects [9,10]. In concordance to the hypothesis, previous study using high-fat diet (HFD)-induced experimental model demonstrated that healthy FDR subjects gained more weight and experienced greater IR measured by HOMA-IR, albeit no significant difference was observed in lipid profile compared to the control group [11]. Meanwhile, studies on the role of inflammation in HFD-associated induction of IR in FDR subjects have shown mixed results. One study demonstrated increase in inflammatory markers in both FDR and non-FDR subjects similarly [12], while in another, HFD only induced alteration in endothelial markers [13]. Multiple factors might modulate the response to these conflicting results, including subject ethnicity [14], gender, macronutrient content, and dietary pattern given [9].

Previous study has demonstrated that a 5-day HFD challenge induced similar IR level and inflammatory response of subjects with the same ethnicity but different rural and urban living background [15]. These results brought us to apply similar HFD experiment on FDR subjects living in urban area to investigate whether the hereditary background might influence the response thus obtained different results. As multiple abnormalities related to lipid metabolism are frequently occurred within FDR subjects at early age [3,4], we hypothesized that healthy FDR subjects would have greater IR and inflammation effect when induced by a 5-day HFD intervention compared to the non-FDR subjects.

## 2 Methods

### *2.1 Study participants*.

Participants recruited in this study were divided into 2 groups. The FDR group consisted of male offspring of T2DM patients visiting the outpatient diabetes clinic at Dr. Cipto Mangunkusumo Hospital, Jakarta, Indonesia. Non-FDR served as the control group composed of offspring of hospital employee or relatives who did not have any family history of T2DM in first- and second-degree, matched for age-sex-body mass index (BMI) to those in FDR group. Inclusion criteria were healthy males, aged between 25-39 years, whose BMI of 18-34.9 kg/m^2^, normoglycemic and normotensive as determined respectively by HbA1c ≤5.6%, fasting blood glucose test being <100 mg/dl, and blood pressure of <140/90 mmHg [16]. Individuals happened to have recent infections, smoking history, taking any medications that could affect inflammation or IR, were excluded from the study.

### 2.2 Screening test

**Figure S1.**
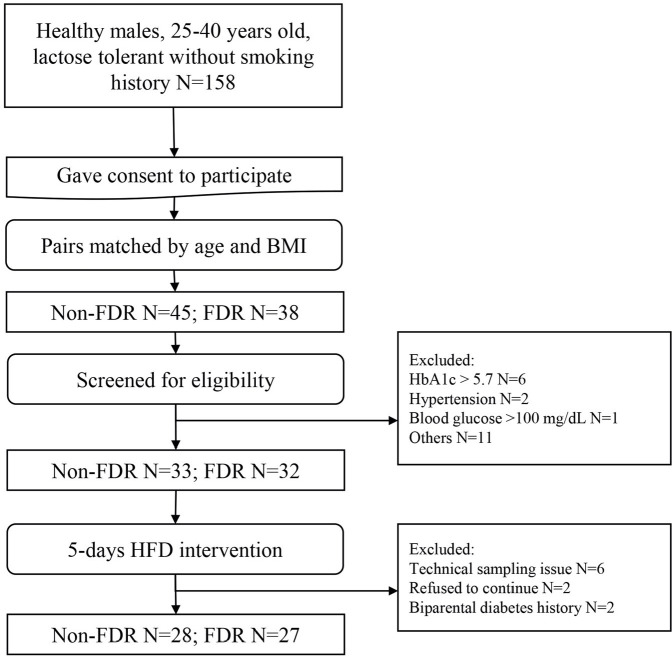
Subject recruitment workflow. BMI, body mass index; FDR, first-degree relatives; HFD, high-fat diet.

Subjects were measured for BMI and waist circumference (WC). Clinical parameters screened were fasting blood glucose which was carried out with glucometer (AccuCheck, Roche) using capillary blood, point-of-care HbA1c level using Glycohemoglobin Analyzer EZ test 2.0 system (BioHermes Biomedical Technology Co., Ltd., Wuxi, China), and blood pressure using digital sphygmomanometer (HEM-7121, Omron Healthcare Co, Ltd, Kyoto, Japan) in 2 measurements with 5-minutes interval. Subjects whose screening results match inclusion criteria proceeded with dietary intake recall prior to HFD intervention. A general overview of study workflow was described in supplemental figure S1.

### 2.3 Intervention

A 5-day HFD intervention experiment was designed as previously described with some modifications [15]. In brief, after baseline measurement, each subject received five individual packs of 250 ml dairy cream (Greenfields^TM^ whipping cream, Greenfields Indonesia Ltd, Jakarta, Indonesia). Subjects were instructed to add one pack of cream [900 kcal/day, 100% fat, 0% protein, 0% carbohydrate] per day on top of their regular diet for five consecutive days. The calories from fat in our HFD challenge was considered as the lowest in comparison with similar previous study on Caucasians [17], on both Caucasians and South Asians [14], and on one of Indonesian ethnicity (Floresians) populations [15], by which the additional energy that derived from fat were 60%, 55%, and 56%, respectively. Participants were asked to maintain their daily activities as usual during the HFD intervention.

We also performed 24-hour food recall interviews which were conducted before and after the HFD intervention to assess dietary intake. The food recall before and after HFD was done in 3 days each, which consisted of 2 days in weekdays and 1 day in the weekend. The food recall interview and analysis done before and after HFD were performed by a trained dietician from Southeast Asian Ministers of Education Organization (SEAMEO) Regional Centre for Food and Nutrition (RECFON), Jakarta, Indonesia. In addition to this, we also provided daily assistance via online live video chat as well as food diary record book to maintain regular diet and participants’ compliance to procedure during HFD intervention.

### 2.4 Plasma glucose, lipid, and cytokines measurement

Metabolic and cytokine profile was obtained from venous blood sample drawn one day before starting the HFD intervention (D-0) and one day after the HFD intervention completed (D-6). Blood samples drawn from selected participants were collected into non-additive and EDTA tube (BD vacutainer) to obtain serum (10 mL) and plasma (5 mL), respectively. Prior to blood-drawing, each participant was requested to have an overnight fasting for at least 10-12 hours.

The fasting blood glucose, total cholesterol, high-density lipoprotein (HDL) cholesterol and triglycerides (TGL) were measured on serum using cartridge type IVT-PT03 on a portable automatic LABGEO PT10 analyzer according to manufacturer’s instructions for each analyte. Low-density lipoprotein (LDL) cholesterol (mg/dL) was calculated using the Friedewald formula, [LDL-chol (mg/dL) = [(Total chol)-(HDL-chol)-(TGL/5)]. Serum insulin level was determined by a solid-phase, enzyme-labeled chemiluminescent immunometric assay. Insulin resistance, measured as HOMA-IR was calculated as fasting insulin level (µUI/ml) x fasting plasma glucose (mg/dL) all divided by 405[17]. All parameters mentioned above were assessed at Department of Clinical Chemistry and Laboratory Medicine Cipto Mangunkusumo Hospital, which is ISO 15189:2012 accredited.

Cytokines widely recognized to be associated with chronic low-grade inflammatory status, including pro-inflammatory cytokines TNF-α, IL-1β, IL-6, and IFN-γ as well as their corresponding anti-inflammatory cytokines, IL-10, IL-1RA, and IL-4 were measured on customized human multiplex immunoassay kit (Magnetic Luminex Assay, Cat. No LXSAHM) and read using a Luminex 200 according to the manufacturer’s instructions. Plasma cytokines concentrations were then calculated using a 5-standard parameter logistic that fit on xPONENT for LX 100/200 version 4.2 Build 1509 software.

### 2.5 Statistical analysis

Sample size was calculated based on previous HFD intervention study that demonstrated significant changes of HOMA-IR at 0.5 SD using n=17 subjects per group [15]. Using power of 90% and alpha of 0.05, the sample size was estimated to be as minimum as 19 subjects per group to achieve similar impact. Data analysis was carried out using SPSS for Windows version 20.0 (IBM). Variables that did not meet the assumption of normality based on Shapiro-Wilk test and Q-Q plots were log transformed or were further classified as non-normally distributed data. Normally distributed data were presented as mean±standard deviation while non-normally distributed data were presented as median (interquartile). To assess the difference of HFD effects on variables within and between group, Student’s paired or independent t-tests were applied on normally distributed data and non-parametric tests (Wilcoxon signed-rank test and Mann-Whitney) was applied on non-normally distributed data, respectively. Statistical significance was set at p<0.05 within 95% confidence of interval.

## 3 Results

Sixty-five male participants were recruited to undergo HFD intervention. From these number we eventually got 28 non-FDR and 27 FDR subjects by the end of HFD intervention due to technical sampling issues (six participants), subjects withdrawn their participations during trial (two subjects), and two were excluded for biparental T2DM history. Biparental T2DM history have three times higher risk of getting T2DM compared to individuals with single parental history thus might result in bias [18]. There were no unintended adverse events.

### 3.1 Comparison of dietary intake at baseline and after HFD intervention

At baseline level, both groups used carbohydrate as their main energy source, followed by fat and protein. Both groups also already shown a HFD dietary pattern prior to HFD intervention, based on their fat percentage above 35% of total calories intake [19]. During HFD intervention, under daily monitoring from our team, both groups showed good compliance to consume all provided heavy cream and maintained their regular diet unchanged. As a result, approximately 60% higher total caloric intake (p <0.05) was observed after HFD intervention in both groups. Of total caloric intake from HFD intervention, the approximately 60% caloric intake was derived from fat, so it could be assumed that an acute HFD environment compared to their basal dietary profile had successfully been created. In addition to this, our HFD intervention induced alteration of basal macronutrient composition from carbohydrate as their main energy source to fat. Reported dietary intakes at baseline and after HFD intervention are given in Table 1.

**Table 1.** Comparison of dietary intakes at baseline and after HFD intervention

	Non-FDR (n=28)			FDR (n=27)			p value between group^b^	
	Baseline	After HFD	p-value^a^	Baseline	After HFD	p-valuea	Baseline	After HFD
Energy (kkal)	1484±416	2384±416	<0,005	1580±298	2489±251	<0,005	0.26	0.09
Protein (% total)	14±3.5	9±0.3	<0,005	15±2.5	10±0.4	<0,005	0.09	0.85
Carbohydrate (% total)	53±7.8	32±0.8	<0,005	49±9.4	31±0.8	<0,005	0.1	0.59
Lemak (% total)	35±4.5	60±0.9	<0,005	39±9.1	61±0.9	<0,005	0.05*	0.1

FDR, first-degree relatives. Data were presented as mean±standard deviation. Analysis of difference between after HFD and baseline level were assessed using ^a^Paired Student t-test (p <0.05) whilst analysis of difference in changes between FDR and non-FDR group was performed using ^b^independent Student’s t-test (p <0.05).

### 3.2 Comparison of metabolic profiles at baseline and after HFD intervention

Although BMI was being matched and showed no significant difference, however we found that FDR subjects were more centrally obese compared to nonFDR subjects (89.6±10.5 cm vs 80.3±9.3 cm; p=0.001) (Table 2). At this point, we found that increase in WC was correlated with increasing fasting insulin (r=0.5, p<0.001), HOMA-IR (r=0.5, p<0.001), TGL (r=0.4, p=0.002), and LDL (r=0.3, p=0.02) in total subjects, while HDL level was conversely correlated with increasing WC (r=-0.3, p=0.03) (Supplemental Table S1).

**Table 2.** Subject characteristics

	Non-FDR (n=28)	FDR (n=27)	*p* value
Age (years)	29.1±4.3	29±3.4	0.95
Body mass index (kg/m^2^)	23.6±3.5	25.3±3.8	0.08
WC (cm)	80.3±9.3	89.6±10.5	0.001*
Systole (mmHg)	122.6±7.6	121.6±9.2	0.64
Diastole (mmHg)	80±7.1	82±6.8	0.3
HbA1c (%)	5.1±0.3	5.2 ± 0.3	0.64

FDR, first degree relatives. Data are presented as mean±standard deviation and frequencies for ethnicity and parental history. Statistical significances were calculated using unpaired Student’s t-tests. *p<0.05

As seen in Table 3, FDR subjects had higher mean level of fasting insulin (8.2±2.6 µIU/mL vs 7.2±2.5 µIU/mL), fasting glucose (83.9±6.6 vs 83±8.7 mg/dL), HOMA-IR [1.8 (1.5) vs 1.5 (0.7)], total cholesterol (197.7±36.7 mg/dL vs 187.0±39.0 mg/dL), TGL [105 (66) mg/dL vs 78.5 (50.5) mg/dL], and LDL (142±34.2 mg/dL vs 130.7±36.2 mg/dL), but with lower HDL level (44.8±8.2 mg/dL vs 47.9±6.8 mg/dL) compared to nonFDR subjects. However, we did not find any significant differences between group in those variables except for the TGL level.

**Table 3.** Comparison of metabolic response toward HFD intervention

	Non-FDR (n=28)				FDR (n=27)				p value between group	
Variables	Baseline	After HFD	*p value* ^b^	Fold changes From baseline level	Baseline	After HFD	*p value* ^b^	Fold changes to baseline level	At Baseline level^a^	Fold changes from baseline level^b^
**Fasting glucose** **(mg/dL)**	83.0±8.7	82.9±8.8	0.93	0±0.1	83.9±6.6	85.9±9.6	0.3	0.002±0.1	0.56	0.33
**Fasting insulin** **(µIU/ml)**	7.2±2.5	8.4±3.2	0.03*	0.23±0.4	8.2±2.6	10±4.3	0.02*	0.3±0.5	0.29	0.76
**HOMA-IR**	1.5 (0.7)	1.6 (0.8)	0.07	0.25±0.5	1.6 (0.8)	1.8 (1.5)	0.02*	0.2 (0.7)	0.14	0.79
**Total** **cholesterol** **(mg/dL)**	187.0±39.0	188.0±35.6	0.8	0.02±0.1	197.7±36.7	211±39.2	0.01*	0.1±0.1	0.45	0.09
**TGL (mg/dL)**	78.5 (50.5)	85 (59)	0.11	0.17±0.5	105 (66)	113 (69)	0.8	-0.04 (0.4)	0.03*	0.25
**HDL-c (mg/dL)**	47.9±6.8	49.8±8.0	0.12	0.04±0.1	44.8±8.2	48.8±9.5	<0.001**	0.09±0.1	0.1	0.18
**LDL-c (mg/dL)**	130.7±36.2	127.8±33.8	0.5	0±0.2	142±34.2	148.1±35.2	0.15	0.06±0.2	0.39	0.18

FDR, first-degree relatives; HFD, high-fat diet; HOMA-IR, homeostatic model assessment of IR; TGL, triacylgliceride; HDL-C, high-density lipoprotein cholesterol; LDL-C, low-density lipoprotein cholesterol. The estimated difference in changes was calculated as fold changes: (after HFD/baseline level)-1. All variables are presented as mean±standard deviation, however, HOMA-IR and TGL levels are presented as median (interquartile). ^a^The differences at baseline level and the difference in changes between group were analyzed using independent t-test or Mann Whitney (HOMA-IR and TGL). ^b^The difference after HFD intervention compared to baseline level within group were analyzed using paired t-test or Wilcoxon paired test (HOMA-IR and TGL). Result are significant if *p<0.05, **p<0.005

Intervention with 250 ml of high-fat dairy cream per day for five consecutive days was able to reduce systemic insulin sensitivity marked by increase of HOMA-IR significantly in FDR group (p=0.02) and a trend to increase in the non-FDR group (p=0.07) (Table 3). This markedly changes of HOMA-IR was mainly driven by the significant increase in fasting insulin levels in both FDR (p=0.02) and non-FDR group (p=0.03) while fasting glucose level remained unaffected by HFD intervention. Concomitantly, we observed a significant increase in total cholesterol (p=0.01) and HDL level (p<0.001) within FDR group whereas the non-FDR group did not show any difference in any lipid variables following HFD intervention. However, we did not observe any significant difference of changes (fold changes to baseline) between group neither for HOMAIR, TGL, nor other metabolic variables observed in this study (Table 3).

### 3.3 Comparison of plasma cytokine changes in response to HFD

At baseline, both groups displayed similar pro-inflammatory plasma cytokines level (Table 4). Following HFD intervention, we observed significant upregulation of plasma IL-6 level in both FDR [from 1.8 (0.2) pg/mL to 2.5 (2) pg/mL, p<0.001] and non-FDR group [1.82 (0.2) pg/mL to 2.3 (1) pg/mL, p=0.001]. On the other hand, plasma TNF-α and IL-1β level in FDR group were slightly increased [from 15.4 (7.8) pg/mL to 17.8 (10.9) pg/mL, p=0.13; from 2.9 (1.1) pg/mL to 3.5 (0.8) pg/mL, p=0.84; respectively]. Meanwhile, nonFDR group showed a slightly decrease in plasma TNF-α level [from 15.4 (6.2) pg/mL to 14.7 (5.8) pg/mL, p=0.9] and stable IL-1β level at 2.9 (0.8) pg/mL. Plasma IFN-γ in FDR group remain unaffected at 0.9 (0.2) pg/mL whilst the non-FDR group showed a trend to decrease [from 0.9 (0.2) pg/mL to 0.8 (0.1) pg/mL, p=0.09]. With regards to the anti-inflammatory cytokines, we observed a significant increase of plasma IL-1RA after HFD intervention only in FDR group [from 4.5 (2.4) pg/ mL to 5.2 (2.4) pg/mL, p=0.03] while in the non-FDR group it remained stable. The HFD intervention also made slight increase on IL-10 plasma on both FDR [from 8.3 (2.4) pg/mL to 8.7 (2.4) pg/mL, p=0.57] and non-FDR group [from 7.9 (2.1) pg/mL to 8.5 (2.1) pg/ mL, p=0.65]. However, the plasma IL-4 level seemed to be unaffected by HFD intervention in both FDR (p=0.2) and non-FDR (p=0.12) groups. Although HFD intervention caused alteration in some of cytokines examined, we did not find any significant difference in term of changes pattern between FDR and non-FDR group.

**Table 4.** Comparison of plasma cytokines level at baseline and after HFD intervention

	Non-FDR (n=28)				FDR (n=27)				p value between group	
Variables	Baseline	After HFD	*p value* ^b^	Fold changes From baseline level	Baseline	After HFD	*p value* ^b^	Fold changes to baseline level	At Baseline level^a^	Fold changes from baseline level^b^
**TNF-α (pg/mL)**	15.4 (6.2)	14.7 (5.8)	0.90	-0.02 (0.3)	15.4 (7.8)	17.8 (10.9)	0.13	0.05 (0.2)	0.95	0.2
**IL-1β (pg/mL)**	2.9 (0.8)	2.9 (0.8)	0.38	0 (0.2)	2.9 (1.1)	3.5 (0.8)	0.84	0 (0.5)	0.67	0.73
**IL-6 (pg/mL)**	1.8 (0.2)	2.3 (1)	0.00**	0.29 (0.7)	1.8 (0.2)	2.5 (2)	0.00**	0.4 (1.1)	0.11	0.96
**IFN-γ (pg/mL)**	0.9 (0.2)	0.8 (0.1)	0.09	-0.07 (0.1)	0.9 (0.2)	0.9 (0.2)	0.81	0 (0.2)	0.86	0.16
**IL-10 (pg/mL)**	7.9 (2.1)	8.5 (2.1)	0.65	0 (0.2)	8.3 (2.4)	8.7 (2.4)	0.89	0 (0.4)	0.91	0.65
**IL-1RA (pg/** **mL)**	4.5 (1.9)	4.5 (1.9)	0.96	0 (0,2)	4.5 (2.8)	5.2 (2.8)	0.03*	0.1 (0.2)	0.3	0.1
**IL-4 (pg/mL)**	0.8 (0.2)	0.8 (0.3)	0.12	0 (0.3)	0.1 (0.3)	0.1 (0.2)	0.2	0 (0.1)	0.09	0.08

FDR, first degree relatives; HFD, high-fat diet. The estimated difference in changes was calculated as fold changes: (after HFD/baseline level)-1. All variables are presented as median (interquartile). ^a^Wilcoxon paired test, ^b^Mann Whitney. *p<0.05, **p<0.005

In terms of immune response polarization, the ratio between pro-inflammatory cytokines and their corresponding anti-inflammatory cytokines were used to determine the type of immune response. The ratio of TNF-α/IL-10, IL-1β/IL-1RA, and IFN-γ/IL-4 were used to measure the polarization responses after HFD intervention. We found that FDR responded to HFD intervention with a significant increase in TNF-α/IL-10 ratio [from 1.7 (0.6) to 2 (0.7), p=0.03] while nonFDR tend to decrease [from 2 (0.9) to 1.7 (0.6), p=0.17] (Table 5). This finding was only observed in TNF-α/IL-10 ratio, as other pro- to anti-inflammatory cytokine parameters did not show any difference between groups. Analysis on the fold changes of TNF-α/IL-10 ratio further confirmed that FDR group responded the HFD intervention differently compared to nonFDR group in TNF-α/IL-10 ratio [0.1 (0.3) vs -0.1 (0.2), p<0.01] (Table 5).

**Table 5.** Comparison of plasma pro-/anti-inflammatory cytokines at baseline and after HFD intervention

	Non-FDR (n=28)			FDR (n=27)			p value between group	
	Baseline	After HFD	p value^a^	Baseline	After HFD	p value^a^	p value^a^	Fold changes from baseline level^b^
**TNF-α/IL-10**	2 (0.9)	1.7 (0.6)	0.17	1.7 (0.6)	2 (0.7)	0.03*	0.73	<0,01*
**IL-1β/IL-1RA**	0.7 (0.2)	0.6 (0.2)	0.27	0.7 (0.3)	0.6 (0.2)	0.35	0.37	0.48
**IFN-γ/IL-4**	1 (0.3)	0.9 (0.3)	0.11	0.9 (0.2)	0.9 (0.2)	0.35	0.49	0.07

FDR, first degree relatives; HFD, high-fat diet. The estimated difference in changes was calculated as fold changes: (after HFD/baseline level)-1. All variables are presented as median (interquartile). ^a^Wilcoxon paired test, ^b^Mann Whitney. *p<0.05, **p<0.005

### 3.4 Association between FDR status and elevated plasma TNF-α/IL-10 ratio

**Figure 1.**
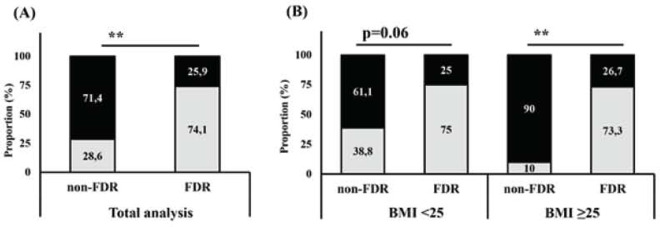
Association between FDR status and elevated plasma TNF-α/IL10 in response to HFD. Association was assessed using Pearson Chi-square test of independence based on whole group or BMI stratification. The proportion of subjects with elevated plasma TNF-α/IL-10 response was significantly different between group if *p<0.05, **p<0.005; not significant (ns) if p>0.05

Since we found a significant difference in TNF-α/IL-10 ratio changes towards HFD-intervention between groups, we then performed Pearson Chi-square and Mantel Hazel odds ratio test to determine the association between this typical TNF-α/IL-10-related pro-inflammatory response with the FDR status. The result was further confirmed that this marked increase of TNF-α/IL-10 ratio was associated with the FDR status since it occured in 20 out of 27 FDR subjects while on the other hand, this typical response was found in only 8 out of 28 non-FDR subjects (p=0.01) (Table 6). Further analysis based on BMI stratification consistently demonstrated the higher proportion of FDR subjects with elevated plasma TNF-α/IL-10 ratio (Figure 1). The odds ratio for FDR subjects to develop increased TNF-α/IL-10 ratio towards HFD intervention was 7.1 (95% CI 2.2–23.4) compared to non-FDR subjects and became 24.8 (95% CI 2.3–262.6) when BMI was stratified to >25 kg/m2 (Table 6).

**Table 6.** Association analysis between FDR status and TNF-α/IL-10 ratio response in overall subjects and stratified by BMI

Stratification	TNF-α /IL-10 ratio	Frequency		p value^a^	Estimated Odds Ratio^b^
		Non-FDR	FDR		
Total samples	No increase Increase	20 (71.4%) 8 (28.6%)	7 (25.9%) 20 (74.1%)	0.001**	7.1
BMI <25 kg/m^2^	No increase Increase	11 (61.1%) 7 (38.9%)	3 (25%) 9 (5%)	0.06	4.7
BMI ≥25 kg/m^2^	No increase Increase	9 (90.0%) 1 (10.0%)	4 (26.7%)11 (73.3%)	0.008*	24.8

FDR; first-degree relatives; BMI, body mass index. The magnitude of TNF-α /IL-10 ratio changes following HFD intervention was calculated as TNF-α /IL-10 ratio after HFD divided by TNF-α /IL-10 at baseline minus 1. It was determined as increased if the value were positive, and stagnant/decreased if the value were 0 or negative. ^a^Chi square test, ^b^Mantel Hazel odds ratio of having risk to develop increase TNF-α / IL-10; *p <0.05, **p<0.05

## 4 Discussion

In the present study, we showed that a 5-day HFD intervention was able to increase systemic IR, cholesterol, and plasma IL-6 level in healthy subjects living in urban area in Indonesia. The FDR subjects in this study were tend to have higher increase in all metabolic and inflammation cytokine level which was mainly mediated by their higher BMI compared to the non-FDR, suggesting the adiposity level contribute to accelerate the IR process. In addition to this, HFD intervention was significantly altered the immune polarization toward a proinflammatory response in FDR group based on increase in plasma TNF-α/ IL-10 ratio, while in contrary, a protective response characterized by decrease in the TNF-α/IL-10 ratio was shown in the non-FDR group. Moreover, FDR subjects had an OR of 7.1 (95% CI 2.2-3.4) to develop elevated plasma TNF-α/IL-10 ratio in response to HFD. When the analysis was separated by BMI, the tendency was as high as 24.8 (95% CI 2.3-262.6) in FDR subjects with BMI ≥25 compared to non-FDR counterpart, suggesting the tendency of having higher lipid accumulation contributed to their propensity when facing HFD environment.

This study recruited healthy participants with normoglycemic conditions and paired FDR and non-FDR subjects based on similar aged-BMI criteria. However, indications of reduced insulin sensitivity have already shown by our FDR subjects prior to HFD intervention as we observed higher fasting insulin level and HOMAIR compared to the non-FDR subjects. In addition to this, our FDR subjects demonstrated slight alteration in three components of IR-related dyslipidemia markers which were higher TGL, higher LDL, and lower HDL [20], compared to non-FDR group. Reduced insulin sensitivity accompanied by dyslipidemia were also reported by other studies which observed healthy males in Jakarta [3], white young males [21], and Asian healthy adults [22-24]. Furthermore, the alteration in lipid profiles found within our FDR subjects were accompanied by significantly bigger WC compared to that of non-FDR subjects (p=0.001). Large WC has long been known to be associated with the risk of T2DM and a marker of visceral fat accumulation [6,25]. Many population-based studies previously had provided evidence that FDR have bigger WC compared to general population [3,5,22-25]. Alteration in lipid metabolism might explained this phenomenon [1]. Individuals with family history of T2DM were shown to have larger subcutaneous adipose tissue for a given BMI and reduced glycolysis rate in the skeletal muscle cells which are correlated with higher ectopic fat accumulation compared with individuals without family history of T2DM [1,2]. Furthermore, one study also reported previously that incidence of multiple metabolic abnormalities, including central obesity and alteration in lipid profile, were increased in young FDR subjects in Indonesia [5]. Taken together, our FDR subjects most likely represented the common characteristics of having higher IR markers compared to non-FDR subjects before HFD intervention.

Low-grade inflammation has been linked to IR and proposed as one of the earliest findings in the pathogenesis of T2DM [6,27]. It has been reported that circulating TNF-α was higher in healthy FDR compared to that of non-FDR individuals [7,28], indicating a low-grade inflammation has already occurred within healthy individuals with family history of T2DM. However, there were inconsistent findings which reported no difference in circulating TNF-α and IL-6 in FDR compared to control group [29]. Regarding to these conflicting results, our study did not find any difference in all pro-inflammatory as well as the anti-inflammatory cytokines at baseline level between groups (Table 4). Differences in genetic background of participants between studies might contribute to the inconsistent outcomes thereby predicting the IR risk based on baseline cytokines level would create bias.

High-fat diet intervention has been commonly used to observe subtle metabolic alteration that might already be present in one’s body and becomes detectable when exposed to excessive caloric intake environment [9]. Previous study had demonstrated that a greater IR as a response to a-5 days HFD intervention was shown by Asian ethnicity while in Caucasians counterparts were unaffected [14]. This result was confirmed by another study on Indonesian healthy males, using similar amount of HFD [15]. In line with those results, our HFD intervention was able to increase fasting insulin, fasting glucose, and peripheral IR in both FDR and non-FDR group similarly (Table 3). Although no significant differences in changes were observed, FDR group demonstrated substantially greater increase in fasting insulin, HOMA-IR, total cholesterol, and HDL level following HFD intervention. Our findings, along with other study [11], supported the hypothesis that metabolic alterations associated with HFD would be greater in individuals with parental history of T2DM than those without one. Of notes, this greater metabolic alteration presumably might be as consequences of the higher baseline level of most IR-related parameters found in FDR compared to non-FDR group. Having higher BMI or WC did not seem to affect the metabolic changes, since we did not find any difference in all metabolic parameters following subgroup analysis based on normal BMI (<25 kg/m^2^) and overweight (≥25 kg/m^2^).

In terms of immune responses, it has been established that HFD induced elevation of low-grade inflammation markers, such as TNF-α, IL-1, and IL-6 [30,31]. In this study, we demonstrated that HFD intervention induced significant increase of IL-6 in both groups similarly (Table 4). Interleukin-6 has been known to play important role in glucose metabolism through regulation in insulin signalling pathway [32], thereby it would be plausible to assume that this markedly increase IL-6 was a compensatory result of significant increase in insulin level following HFD intervention. Previous study also supported this hypothesis by demonstrating that hyperinsulinemia induced significant elevation in IL-6 plasma level in both FDR and non-FDR group [33].

Interesting findings were shown by cytokine IL-1β and its corresponding anti-inflammatory IL-1RA. A slight increase in plasma IL-1β was found only in FDR group, while in non-FDR it remained unaffected by HFD challenge (Table 4). The higher increase in IL-1β level as observed by FDR subjects might correlated with their tendency to have bigger WC. As reported by previous study, the level IL-1β in VAT were significantly higher compared with subcutaneous adipose tissue [34], thus presumably bigger WC affect the degree of systemic HFD-induced IL-1β as observed in our FDR subjects. In paralel to this, a significant increase in plasma IL-1RA level were also observed only in FDR subjects as compensatory mechanism following HFD-induced IL-1β secretion. The increase of IL-1RA seems to correlate directly with degree of fat accumulation in VAT, since we found significant correlation between increase in IL-1RA and WC (R=0.52, p=0.047) in our FDR subjects with BMI ≥25. Despite no significant differences found between groups, the results could indicate an alteration in IL-1RA function that makes FDR individuals tend to exhibit stronger compensatory mechanism of IL-1RA to block the IL-1β activity following HFD challenge, than the non-FDR subjects. In agreement with our assumption here, previous studies demonstrated increased plasma level of IL-1RA was found to precede the onset of T2DM [7,35,36].

Elevated TNF-α stimulates the production of IL-10 as counter-regulatory mechanism to maintain homeostasis. Any impairment involving either TNF-α or IL-10 could lead to imbalance of immune response [37]. Individuals with family history of T2DM and T2DM patients have been proposed to have reduced function of IL-10 to suppress TNF-α production based on reduced IL-10 level following hyperinsulinemic-induced condition, as reported previously [33]. Our study provided evidence that alteration in homeostatic mechanism between TNF-α and IL-10 indeed, was occured in FDR subjects. As a response to HFD challenge, our FDR subjects demonstrated an elevated level of plasma TNF-α, while in contrary, the non-FDR group showed a decreased plasma TNF-α (Table 4). The tendency of our FDR subjects to have bigger WC might again contribute to the results because we found significant correlation between changes in plasma TNF-α and bigger WC in FDR subjects with BMI ≥25 (R=0.56, p=0.03). This elevated plasma TNF-α as observed in our FDR subjects was actually accompanied by an elevated plasma IL-10 level comparable to that of non-FDR group, however, such an increase in IL-10 level did not implicate in reduced plasma TNF-α level in FDR group. Impairment in inhibiton function of IL-10 has been proposed as mechanism causing inflammation status in T2DM [38]. In concordance to this hypothesis, our result suggested that FDR subjects might already have reduced suppressive function of IL-10 that cause their inability to compensate the HFD-induced increase in TNF-α level in the same manner with that observed in non-FDR group.

Our findings were further clarified by subsequent analysis using TNF-α/IL-10 ratio as pro-inflammatory index parameter [39] to describe the polarization response occurred. Using this parameter, we observed a significant increase of TNF-α/IL-10 ratio in FDR group following HFD intervention, whilst as expected, a decrease of TNF-α/IL-10 ratio was demonstrated in the non-FDR group. The results proved that although the HFD-induced increase in TNF-α level was not significantly different between groups, the impaired function of IL-10 in FDR subjects was sufficient to shift the systemic immune polarization into a pro-inflammatory environment. Furthermore, we found that the tendency to develop this particular pro-inflammatory response was strongly associated with FDR status as the proportion of subjects that responded HFD with increased plasma TNF-α/IL-10 ratio was significantly higher in the FDR group than that of the non-FDR group (Figure 1). The FDR subjects had an OR of 7.1 to develop elevated TNF-α/IL-10 ratio in response to HFD and an OR of 24.8 when further stratified by BMI ≥25 compared to non-FDR group. Taken together, the data generated from our study provided a unique illustration on how a 5-days HFD intervention induced shifting in homeostatic mechanism between TNF-α and IL-10 resulted in systemic polarization to pro-inflammatory environment in FDR subjects. The mechanism seems to be correlated with increase in lipid accumulation in VAT thus promoting aggravation in IR development.

There are limitations in this study worth for future discussion. First, although the participants were passed through an age-BMI matched selection prior to recruitment, bigger WC was seemed inseparable with FDR group when compared to the control group. Adipose tissue is one of major sources of TNF-α and IL-10 [40], so that bigger WC could contribute to the significant result in our FDR group. Thereby future investigation should carefully include WC as the pair-matched criteria. Second, inflammation was highly dependent with environment milieu. Even though the inclusion criteria were set to recruit healthy subjects, our FDR started the HFD intervention with greater level in most of IR parameters, particularly in TGL parameter, that could enhance inflammation [40]. Lastly, about 75% of IL-10 response was influenced by genetic factors [38], thereby lack of inheritable-related parameters nor functional studies at cellular level makes the result arguable, hence the need of further confirmation.

In conclusion, our study demonstrated that five consecutive days of additional 250 ml high-fat dairy product into dietary meals was sufficient to impair insulin sensitivity and increase plasma IL-6 level among healthy urban Indonesian males. Along with this results, HFD intervention revealed that a shift in the homeostatic mechanism between TNF-α and IL-10 toward a pro-inflammatory response occurred in FDR individuals. The cluster of FDR subjects with BMI ≥25 was most likely to develop a pro-inflammatory response that marked by an elevated plasma TNF-α / IL-10 when facing HFD challenge. This alteration in TNF-α and IL-10 interaction might explain why FDR of T2DM would experience earlier progression to IR-related diseases compared to the non-FDR individuals when facing relatively similar obesogenic environment.

## Figures and Tables

**Table S1.** Correlation analysis between waist circumference and metabolic variables at baseline level

Variables	Waist circumference correlation analysis					
	Total samples		Non-FDR		FDR	
	R	P	R	P	R	P
Fasting glucose	0.1	0.3	0.4	0.03*	-0.2	0.3
HOMA-IR	0.5	0.00**	0.6	0.00**	0.3	0.08
Total cholesterol	0.1	0.2	0.4	0.02*	-0.1	0.54
TGL	0.3	0.01*	0.3	0.12	0.2	0.2
HDL-c	-0.3	0.02*	-0.1	0.5	-0.4	0.06
LDL-c	0.3	0.05	0.4	0.02*	0	1

FDR, first-degree relatives; HOMA-IR, homeostatic model assessment of insulin resistance; TGL, triglyceride; HDL-C, high-density lipoprotein cholesterol; LDL-C, low-density lipoprotein cholesterol. Correlation between waist circumference and metabolic variables were analyzed using Pearson correlation test (*p<0.05, **p<0.005)

## Abbreviations

BMI: Body mass index

FDR: First-degree relatives

HDL: High-density lipoprotein

HFD: High-fat diet

HOMA-IR: Homeostatic model assessment for insulin resistance

IFN: Interferon

IL: Interleukin

IR: Insulin resistance

LDL: Low-density lipoprotein

OR: Odds ratio

SAT: Subcutaneous adipose tissue

TGL: Triglyceride

TNF: Tumor necrosis factor

T2DM: Type 2 diabetes mellitus

VAT: Visceral adipose tissue

WC: Waist circumference
